# Tag-SNPs in Phospholipase-Related Genes Modify the Susceptibility to Nephrosclerosis and its Associated Cardiovascular Risk

**DOI:** 10.3389/fphar.2022.817020

**Published:** 2022-05-02

**Authors:** Luz M. González, Nicolás R. Robles, Sonia Mota-Zamorano, José C. Arévalo-Lorido, José Manuel Valdivielso, Juan López-Gómez, Guillermo Gervasini

**Affiliations:** ^1^ Department of Medical and Surgical Therapeutics, Medical School, Institute of Molecular Pathology Biomarkers, University of Extremadura, Badajoz, Spain; ^2^ Service of Nephrology, Badajoz University Hospital, Badajoz, Spain; ^3^ RICORS2040 Renal Research Network, Madrid, Spain; ^4^ Service of Internal Medicine, Badajoz University Hospital, Badajoz, Spain; ^5^ Vascular and Renal Translational Research Group, UDETMA, IRBLleida, Lleida, Spain; ^6^ Service of Clinical Analyses, Badajoz University Hospital, Badajoz, Spain

**Keywords:** nephrosclerosis, atherosclerosis, cardiovascular risk, single nucleotide polymorphism, phospholipases

## Abstract

Nephrosclerosis patients have a high cardiovascular (CV) risk that is very often of more concern than the renal disease itself. We aimed to determine whether variants in phospholipase-related genes, associated with atherosclerosis and CV outcomes in the general population, could constitute biomarkers of nephrosclerosis and/or its associated CV risk. We screened 1,209 nephrosclerosis patients and controls for 86 tag-SNPs that were identified in the *SCARB1*, *PLA2G4A,* and *PLA2G7* gene loci. Regression models were utilized to evaluate their effect on several clinical parameters. Most notably, rs10846744 and rs838880 in *SCARB1* showed significant odds ratios (OR) of 0.66 (0.51–0.87), *p* = 0.003 and 1.48 (1.11–1.96), *p* = 0.007 for nephrosclerosis risk. *PLA2G4A* and *PLA2G7* harboured several SNPs associated with atherosclerosis measurements in the patients, namely common carotid intima media thickness (ccIMT), presence of plaques, number of plaques detected and 2-years ccIMT progression (significant *p*-values ranging from 0.0004 to 0.047). Eight SNPs in *PLA2G4A* were independent risk factors for CV events in nephrosclerosis patients. Their addition to a ROC model containing classic risk factors significantly improved its predictive power from AUC = 69.1% (61.4–76.9) to AUC = 79.1% (73.1–85.1%), *p* = 0.047. Finally, *PLA2G4A* rs932476AA and rs6683619AA genotypes were associated with lower CV event-free survival after controlling for confounding variables [49.59 (47.97–51.21) vs. 51.81 (49.93–51.78) months, *p* = 0.041 and 46.46 (41.00–51.92) vs. 51.17 (50.25–52.08) months, *p* = 0.022, respectively]. Variability in phospholipase-related genes play a relevant role in nephrosclerosis and associated atherosclerosis measurements and CV events.

## Introduction

Chronic kidney disease (CKD) is defined by abnormalities of the kidney structure and/or function for over 3 months with clinical consequences (Stevens and Levin, 2013). CKD increases the risk of all-cause and cardiovascular (CV) mortality (Ortiz et al., 2014) and has been predicted to be the fifth cause of death worldwide by 2040 (Foreman et al., 2018). Nephrosclerosis in particular, which usually refers to the presence of CKD in a hypertensive and aging patient in the absence of histological confirmation, has an enormous impact on the global CV risk (Robles et al., 2020), which often times is of more concern than the progression of the renal disease itself (Zoccali, 2006).

Plasma lipoprotein-associated phospholipase A2 (Lp-PLA2), encoded by the *PLA2G7* gene, transforms low-density lipoprotein (LDL) into a number of proinflammatory mediators. Increased Lp-PLA2 levels has been observed in vascular endothelial dysfunction, atherosclerotic plaque inflammation, and necrotic core formation in plaques, which has led to regard Lp-PLA2 as a predictive marker for atherosclerosis-related CV diseases (CVD) and a promising therapeutic target (Huang et al., 2020). A number of *PLA2G7* single nucleotide polymorphisms (SNPs) have been associated with Lp-PLA2 activity (Suchindran et al., 2010; Grallert et al., 2012) and atherosclerosis (Santoso et al., 2017). The activity of Lp-PLA2 has also been linked to the presence of genetic variants in the human scavenger receptor class B type (*SCARB1*) gene (Manichaikul et al., 2018), a primary receptor for selective transportation of cholesterol for high-density lipoprotein (HDL) and also a typical locus for CVD (Ma et al., 2018; Manichaikul et al., 2018; Koenig et al., 2021). In addition, calcium-dependent cytosolic phospholipase A2α (cPLA2α), a product of the *PLA2G4A* gene, is activated by calcium enabling arachidonic acid release thus leading to the synthesis of various proinflammatory factors (Clark et al., 1991). cPLA2α overexpression has been observed in atherosclerotic arterial wall, mainly in the intima in regions with an inflammatory infiltrate (Elinder et al., 1997) and has recently been revealed as a key factor for calcification in aortic valve interstitial cell cultures (Bonetti et al., 2020).

Our goal was to determine whether genetic variability in three candidate phospholipase-related genes, namely *PLA2G7*, *SCARB1* and *PLA2G4A*, was associated with atherosclerosis and the occurrence of CV events in a population of nephrosclerosis patients. Additionally, we screened a group of healthy individuals for the same SNPs to evaluate the putative role of these variants as risk factors for nephrosclerosis.

## Subjects and Methods

### Study Subjects

The study comprised 1,209 participants, 493 patients diagnosed with nephrosclerosis and 716 controls, who were obtained from 1) the Nephrology Service at Badajoz University Hospital, 2) the NEFRONA repository, a collection of biological samples from Spanish renal patients (Arroyo et al., 2014) and 3) the Instituto de Salud Carlos III biobank (www.bancoadn.org). All patients had stage 3 or higher CKD, i.e., presented with an estimated glomerular filtration rate (eGFR) below 60 ml/min/1.73 m^2^. These patients were all over 18 years of age and were selected among those with biopsy alterations typical of vascular nephropathy or that met clinical criteria, namely the presence of data indicative of the disease (advanced age, long-standing hypertension, left ventricular hypertrophy, initially mild renal failure and proteinuria below 0.5–1 g/24 h) and the lack of signs of other kidney pathologies. Control subjects had all eGFR >60 ml/min/1.73 m^2^. Exclusion criteria included having experienced a CV event before the beginning of the study, organ transplantation, carotid artery surgery, pregnancy, active infection and life expectancy below 1 year.

All subjects gave written consent for their participation in the study, which had been approved by the Bioethics & Biosafety Committee of the University of Extremadura and the Clinical Research Ethics Committee of the Badajoz University Hospital, and that was carried out in accordance with the Declaration of Helsinki and its subsequent revisions.

### Clinical Variables

An amount of more than 500 mg of protein (or more than 300 mg of albumin) in 24-h urine was defined as proteinuria (patients with proteinuria >1 g were biopsied for diagnosis confirmation). Renal function was estimated using the Modification of Diet in Renal Disease (MDRD) equation. Diagnostic and prognostic stratification of patients was carried out using the CONSORTIUM-CKD equation and the KDIGO classification and table of progression risk (www.kidneyriskfailure.org). CV risk was defined as the likelihood of experiencing a CV event (fatal or not) in a four-year follow-up. Patients were followed up until the earliest of CV event, death, or end of study. CV events included acute myocardial infarction, acute coronary syndrome, coronary catheterization requiring angioplasty, coronary bypass, typical angina with positive stress tests, sudden death, cerebrovascular accident, peripheral arterial disease, aortic aneurisma and lower limb ischemia.

### Arterial Ultrasound

The presence of atheromatous plaques was assessed in 412 out of the 493 nephrosclerosis as described elsewhere (Abajo et al., 2017). In brief, explorations were carried out in ten different arterial territories with a high-resolution B-mode ultrasound (Vivid BT09, GE Healthcare, Waukesha, WI, United States) according to guidelines of the Mannheim IMT Consensus (Touboul et al., 2004) and the American Society of Echocardiography (Stein et al., 2008). In the case of intima media thickness (IMT), the left and right common carotid arteries (CCAs) were explored in the anterolateral, posterolateral, and mediolateral directions. IMT was defined as the distance between the leading edge of the lumen intima echo and the leading edge of the media-adventitia echo in the far wall. It was measured 1 cm from the bifurcation. Three longitudinal measurements of IMT were accomplished on the right and left CCAs. We used the mean of the three right and left measurements in the analysis. Atheromatous plaques were considered when IMT was larger than 1.5 mm protruding into the lumen. Common carotid intima media thickness (ccIMT) progression was calculated for nephrosclerosis patients by subtracting ccIMT at baseline from ccIMT at 24 months for each side. Values were then averaged and expressed in mm changed per year. An atherosclerosis severity score ranging from 0 to 3 was created [0: Ankle Brachial Index (ABI) > 0.9 and ccIMT <90% reference interval; 1: ABI = 0.7–0.9 and/or ccIMT ≥90% reference interval; 2: presence of a carotid plaque with stenosis <125 cm/seg; 3: plaque with stenosis ≥125 cm/seg and/or ABI <0.7]. The ABI is a quick, noninvasive way to check for peripheral artery disease. The test compares the blood pressure measured at your ankle with the blood pressure measured at your arm. A low ankle-brachial index number can indicate narrowing or blockage of the arteries in your legs. A value of less than 0.9 is indicative of peripheral artery disease.

### Genetic Analyses

We retrieved the coding sequence and adjacent 3′- and 5′-UTR regions of the *PLA2G7* (ENSG00000146070; HGNC:9040), *SCARB1* (ENSG00000073060; HGNC:1664) and *PLA2G4A* (ENSG00000116711; HGNC:9035) genes, and identified tag-SNPs (polymorphisms that represent genetic variability in a certain area of the gene locus) with Haploview 4.2. A pair-wise tagging with *r* ^2^ ≥ 0.80 and a 5% threshold for minor allele frequencies (MAF) were established to capture common variants. Several of the tag-SNPs included had been previously related to CV outcomes, namely rs10846744, rs5888, and rs1051931. Overall, 86 SNPs were analyzed: 5 in *SCARB1*, 17 in *PLA2G7*, and 64 in *PLA2G4A*. Supplementary Table S1 lists all the SNPs included in the present study.

Genetic material from controls and cases was purified from whole blood samples obtained from biobanks or directly from participants recruited at the Badajoz University Hospital. DNA purification was carried out by means of standard phenol-chloroform extraction followed by ethanol precipitation or by QIAamp DNA Blood Kits. DNA samples were then stored in plastic vials at 4°C until analysis.

Genotyping analyses were conducted with TaqMan® OpenArray using a customized panel on a QuantStudio™ 12K Flex Real-Time PCR System (Life Technologies, Carlsbad, CA, United States). Quality controls (sample trios from the Coriell Institute Biorepository) were included in all runs. Analyses were carried out at the Centro Nacional de Genotipado-Instituto de Salud Carlos III (CeGen-ISCIII; Madrid, Spain, www.cegen.org).

### Statistical Analyses

Categorical variables were compared with the Chi-square test, whilst quantitative variables were compared by the Mann-Whitney or Kruskal-Wallis tests depending on the number of groups. The influence of the SNPs on the risk of nephrosclerosis and other clinical variables was assessed by regression modelling, adjusting for demographics and classic risk factors, namely age, sex, body mass index, ethnicity, diabetes, blood pressure and CKD stage, as formerly described (Valls et al., 2019; González et al., 2021). Kaplan-Meier curves for evaluating the influence of SNPs on CV events were compared with log-rank tests. Subsequently, a Cox regression procedure was carried out to include the effect of additional relevant covariates for those SNPs with log-rank *p*-values < 0.1. The predictive value of the SNPs regarding the risk of nephrosclerosis was evaluated with Receiving Operating Curves (ROC), which were generated for models with classic risk factors with or without genetic information. The DeLong test was used to detect differences between the area under the curve (AUC) of these models. The statistical power calculation was carried out with Quanto software v. 1.2.4 (USC, Los Angeles, CA, United States) by analyzing the frequency of carriers of allelic variants with an arbitrary effect size of 2.0 and a type-I error of 0.05. With the reported incidence of CKD and the available sample size available (n = 1209), the statistical power of the study to identify genetic associations ranged from 0.939 to 0.996 depending on the specific MAF. Statistical analyses were carried out with the IBM SPSS statistical software (SPSS Inc., Chicago, IL, version 22.0) and the *SNPassoc*, *pROC* and *survival* packages in the R environment.

## Results

Main demographic and clinical characteristics of the study population are listed in Table 1. 50.2% of controls and 67.7% of cases were males. Median (range) age values of controls and cases were, respectively, 60 (21–84) and 66 (24–89) years. As expected, nephrosclerosis patients showed a higher incidence of diabetes, hypertension and dyslipidemia (*p* < 0.0001 in all cases). Regarding biochemistry data, differences in glucose, potassium and cholesterol levels were also statistically significant between cases and controls (*p* < 0.001, *p* < 0.0001 and *p* < 0.0001, respectively).

**TABLE 1:** Demographic, biochemical, and clinical characteristics of the population of study. Median (range) or count (percentage) is shown.

		Controls		Nephrosclerosis		*p*-value
Sex	Males	359	50.2%	334	67.7%	<0.0001
	Females	356	49.8%	159	32.3%	
Age, years		60	(21–84)	66	(24–89)	<0.0001
Ethnicity	Caucasian	709	99.0%	482	97.8%	NS
	Other	7	1.0%	11	2.2%	
Weight, kg		75.15	(43.6–160)	79.95	(38–135)	<0.0001
Body mass index		28.25	(17.24–61.76)	29.34	(18.71–51.73)	<0.0001
Glucose (mg/dl)		97	(56–254)	101	(47–355)	<0.001
Calcium (mg/dl)		9.4	(5.6–10.6)	9.4	(6.2–10.7)	NS
Sodium (mEq/L)		141	(133–149)	141	(128–148)	NS
Potasium (mEq/L)		4.5	(3.5–5.9)	4.7	(3.2–7.5)	<0.0001
Cholesterol, mg/dl		201	(121–322)	180	(76–317)	<0.0001
HDL, mg/dl		52.0	(24.8–137)	47.0	(15.1–132)	<0.0001
LDL, mg/dl		125.0	(35–232.1)	103.0	(26–235)	<0.0001
Diabetes	No	660	92.3%	379	76.9%	<0.0001
	Yes	55	7.7%	114	23.1%	
Hypertension	No	310	43.4%	19	3.9%	<0.0001
	Yes	405	56.6%	474	96.1%	
Smoking	Non-smokers	327	45.7%	203	41.2%	NS
	Former smokers	263	36.8%	202	41.0%	
	Smokers	125	17.5%	88	17.8%	
Dyslipidemia	No	313	64.1%	146	31.7%	<0.0001
	Yes	175	35.9%	314	68.3%	
Creatinine, mg/dl		0.83	(0.50–1.28)	1.68	(0.32–8.17)	<0.0001
eGFR, ml/min		88.78	(12–149)	38.11	(5–60.74)	<0.0001
ACR, mg/g		6.40	(0.01–256)	72.62	(0.01–4,583.89)8	<0.0001

eGFR, estimated glomerular filtration rate; ACR, albumin-to-creatinine ratio; NS, non-significant.

### Nephrosclerosis Risk Analysis

Genotyping was successful in 97.6% of the samples. Mean MAF in the whole study population was 22.6%. Six SNPs significantly differed (*p* < 0.05) from the Hardy-Weinberg equilibrium in the control population and were therefore ruled out from further analyses (Supplementary Table S1).

Under a dominant model (carriers vs. non-carriers), two of the five SNPs studied in the *SCARB1* gene and three more variants in *PLA2G4A* showed significant associations with the risk of nephrosclerosis after controlling for meaningful covariates, namely age, sex, body mass index, ethnicity, diabetes, and blood pressure (Table 2). The two identified *SCARB1* variants, rs10846744 and rs838880, showed *p*-values that were 10-fold lower than those of *PLA2G4A* SNPs, with odds ratios (OR) of 0.66 (0.51–0.87), *p* = 0.003 and OR = 1.48 (1.11–1.96), *p* = 0.007, respectively. None of the *PLA2G7* variants were associated with susceptibility to the disease.

**TABLE 2:** Adjusted risk analysis for the risk of nephrosclerosis.

Gene	SNP	Genotype	Control, n (%)	CKD, n (%)	OR	*p*-value
*SCARB1*	rs838880	T/T	314 (45.5)	235 (49.5)	0.66 (0.51–0.87)	0.003
		T/C-C/C	376 (54.5)	240 (50.5)		
*SCARB1*	rs10846744	G/G	460 (66.7)	285 (60.3)	1.48 (1.11–1.96)	0.007
		G/C-C/C	230 (33.3)	188 (39.7)		
*PLA2G4A*	rs78178583	A/A	611 (85.7)	401 (82.2)	1.58 (1.09–2.29)	0.015
		A/C-C/C	102 (14.3)	87 (17.8)		
*PLA2G4A*	rs72709847	T/T	575 (80.8)	413 (84.8)	0.70 (0.49–0.99)	0.046
		T/A-A/A	137 (19.2)	74 (15.2)		
*PLA2G4A*	rs1569479	T/T	153 (22.1)	133 (28.1)	0.73 (0.53–1)	0.048
		T/G-G/G	538 (77.9)	340 (71.9)		

CKD, nephrosclerosis patients; OR, odds ratios with 95% confidence interval.

### Atherosclerosis Measurements

ccIMT median values were significantly higher in patients [0.77 (0.44–1.51) mm] than in controls [0.71 (0.4–1.28) mm; *p* = 2.23E-07], as it was the presence of atheromatous plaques (79.4% vs. 55.9%; *p* = 3.06E-14), the total number of plaques identified in ten different arterial territories [Median (range) = 3 (0–10) vs. 1 (0–9); *p* = 1.48E-22] and the severity score (mean = 1.77 ± 0.71 vs. 1.36 ± 0.82, *p* = 4.83E-14); overall indicating a far higher incidence of atherosclerosis in the CKD patients compared with control subjects.

**FIGURE 1:**
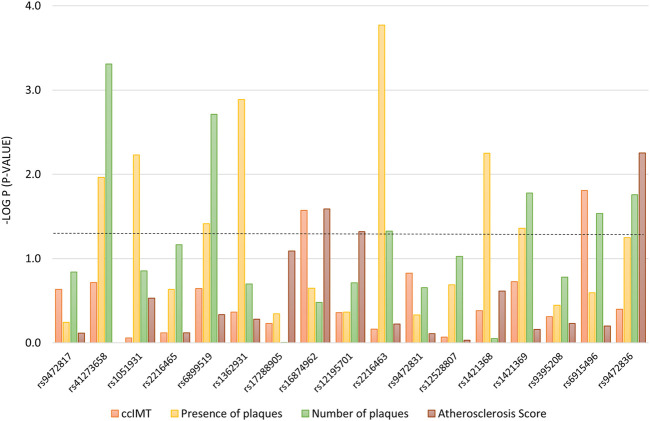
Association between tag-SNPs in the *PLA2G7* gene with atherosclerosis-related measurements. The dotted line denotes the level of statistical significance. ccIMT, common carotid intima media thickness (mm).

The group of nephrosclerosis patients was analyzed to investigate associations with ccIMT, presence of plaques, total number of plaques and atherosclerosis score. *SCARB1* tag-SNPs did not show any relation to atherosclerosis in our study sample (data not shown) after adjusting for confounding variables (age, sex, body mass index, ethnicity, blood pressure, diabetes and CKD stage); however, *PLA2G7* variants were found to particularly affect the presence of atheromatous plaques (7 tag-SNPs) and the number of plaques detected (6 tag-SNPs), with *p*-values ranging from 0.0004 to 0.047 (Figure 1). Six of these variants, namely rs41273658, rs6899519, rs2216463, rs1421369, rs6915496, and rs9472836, were significantly associated with more than one of the four atherosclerosis measurements.

**FIGURE 2:**
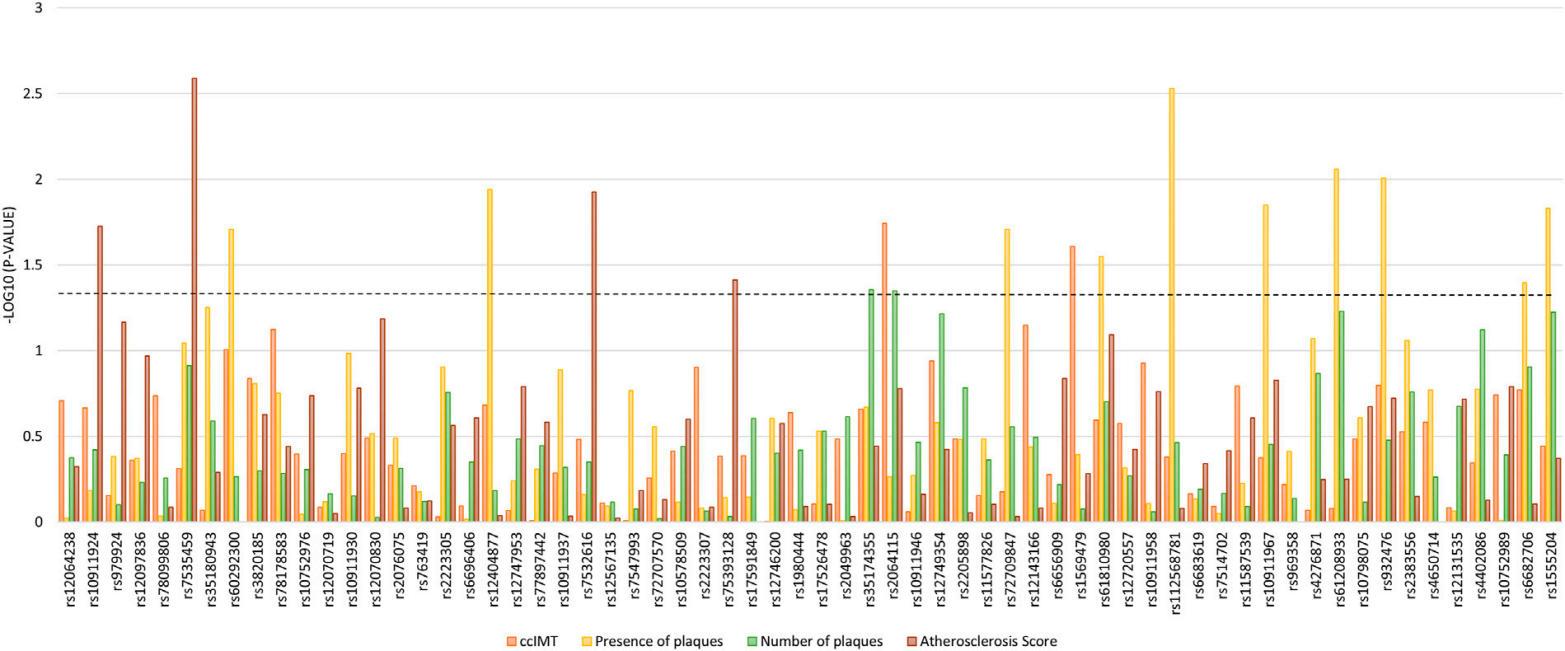
Association between tag-SNPs in the *PLA2G4A* gene with atherosclerosis-related measurements. The dotted line denotes the level of statistical significance.

The impact of variability in *PLA2G4A* is depicted in Figure 2. In this case, observed associations were more noticeable with the variable “presence of atheromatous plaques” and particularly for a distal region of the gene starting at rs72709847, which showed *p*-values between 0.002 and 0.040 (Figure 2).

The median (range) ccIMT progression per year in the nephrosclerosis patients was 0.0125 (-0.22–0.45) mm and the threshold for accelerated progression (75th percentile) was 0.412 mm/year. Six tag-SNPs (one in *PLA2G7* and five in *PLA2G4A*) had a significant impact on the risk of accelerated progression in nephrosclerosis patients after adjusting for confounding variables. Most notably, patients who carried the *PLA2G7* rs9472836-A variant allele showed the strongest association with lower risk of progression [OR = 0.39 (0.19–0.80), *p* = 0.006, Table 3].

**TABLE 3:** Adjusted association analysis with the risk of accelerated ccIMT progression in nephrosclerosis patients.

Gene	SNP	Genotype	SP, n (%)	AP, n (%)	OR	p
*PLA2G7*	rs9472836	G/G	145 (68.4)	60 (84.5)	0.39 (0.19–0.80)	0.006
		G/A-A/A	67 (31.6)	11 (15.5)		
*PLA2G4A*	rs72707570	G/G	85 (41.3)	19 (27.1)	2.0 (1.08–3.69)	0.024
		G/C-C/C	121 (58.7)	51 (72.9)		
*PLA2G4A*	rs10578509	T/T	152 (76.4)	58 (86.6)	0.47 (0.21–1.03)	0.045
		T/del-del/del	47 (23.6)	9 (13.4)		
*PLA2G4A*	rs12746200	A/A	161 (80.1)	61 (89.7)	0.44 (0.19–1.05)	0.049
		A/G-G/G	40 (19.9)	7 (10.3)		
*PLA2G4A*	rs12143166	A/A	70 (34.8)	33 (49.3)	0.54 (0.31–0.-97)	0.039
		A/G-G/G	131 (65.2)	34 (50.7)		
*PLA2G4A*	rs112568781	A/A	152 (75.2)	43 (63.2)	1.84 (1.01–3.34)	0.048
		A/G-G/G	50 (24.8)	25 (36.8)		

SP, slow progression; AP, accelerated progression; OR, odds ratios with 95% confidence interval.

### Parameters of Renal Damage and Function

A mild but significant inverse correlation between ccIMT and eGFR values was observed in the whole population of study (r = -0.181, *p* = 2.15E-07) (Supplementary Figure S1). The genetic association analysis in the nephrosclerosis cohort revealed a central region of the *PLA2G4A* gene locus, tagged from rs10578509 to rs17526478 (positions 1:186873127-186882025) that displayed the most significant associations with glomerular filtration and proteinuria values (Supplementary Figure S2). Remarkably, rs2223307 and rs17591849 SNPs were linked to both parameters (*p*-values for associations with eGFR and albumin-to-creatinine ratios (ACR) were 0.033 and 0.042 for the rs2223307 and 0.036 and 0.016 for rs17591849). Of the remaining SNPs studied, only one variant in *SCARB1* and another in *PLA2G7* were associated with renal function (Supplementary Table S2).

### Cardiovascular events

The 1,209 participants in the study were subjected to follow-up for 4 years (median = 47 months, range = 7–54). Forty-one CV events (8.3%) were reported for nephrosclerosis patients vs. only nine (1.3%) for control subjects [OR = 7.13 (3.4–14.8), *p* = 1.32 e-09]. Table 4 lists the main demographic and clinical characteristics of patients with or without CV events.

**TABLE 4:** Demographic, biochemical and clinical characteristics of nephrosclerosis patients with or without cardiovascular events (CVE) recorded during the four-year follow-up.

	*No CVE*	*CVE*	*p*
Patients	452	41	
Men	300 (66.4%)	34 (82.9%)	0.02
Women	152 (33.-6%)	7 (17.1%)	
Age (years)	66 (24–89)	67 (53–78)	0.01
Weight (kg)	79.8 (38–135)	81.4 (52–125)	0.56
Body mass index	29.4 (18.71–51.73)	29.7 (20.31–48.83)	0.55
Waist circumference (cm)	100 (67–138)	104 (69–131)	0.36
Tobacco			
Nonsmokers	190 (42%)	13 (31.7%)	0.22
Former smokers	185 (40.9%)	17 (41.5%)	
Current smokers	77 (17%)	11 (26.8%)	
Hypertension	435 (96.2%)	39 (95.1%)	0.72
Diabetes Mellitus	100 (22.1%)	14 (34.1%)	0.08
Dyslipidemia	284 (67.8%)	30 (73.2%)	0.47
Chronic kidney disease			
Stage 5D	57 (12.6%)	6 (14.6%)	0.93
Stage 4–5	113 (25%)	10 (24.4%)	
Stage 3	282 (62.4%)	25 (60.9%)	
Biochemical findings			
Cholesterol (mg/dl)	181 (76–317)	165 (108–317)	0.06
Glucose (mg/dl)	100.5 (47–355)	102 (56–195)	0.13
eGFR (MDRD4)	38.2 (5–60.74)	37.4 (13–59.6)	0.7
Albumin/Cr (mg/g)	68.9 (0.01–4,391.65)	173.6 (0.21–4,583.89)	0.17

**FIGURE 3:**
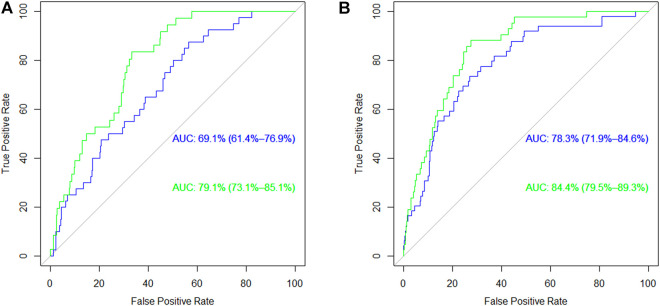
Receiving Operating Curves for the risk of cardiovascular events in **(A)** nephrosclerosis patients and **(B)** the whole population of patients and controls. The blue line corresponds to the model with classic risk factors and the green line corresponds to the same model when genetic information is added. AUC, area under the curve.

The results of genetic association analyses on the incidence of CV events in the nephrosclerosis patients under dominant and recessive inheritance models are shown in Supplementary Table S3. While *SCARB1* and *PLA2G7* did not harbor any relevant SNPs, eight homozygous variant genotypes in the *PLA2G4A* gene were found to significantly affect CV risk (*p*-values ranging from 0.002 to 0.049). We then carried out ROC analysis to establish the predictive power of these eight variants for CV risk. Figure 3A depicts the curves corresponding to predictive models including classic risk factors only, genetics only and both combined in the patients group. Interestingly, AUC values for the classic and genetics only curves were quite similar (*p* = 0.972), whilst the addition of both types of information resulted in a higher AUC of 79.1% (73.1–85.1) that was significantly improved compared to the classic model (*p* = 0.047). When we extended the analysis to both controls and patients (Figure 3B), the classic risk model gained more predictive power [AUC = 78.3% (71.9–84.6)]. This resulted in a high AUC of 84.4% (79.5–89.3) for the combined model but that was not significantly different from the one containing exclusively classic risk factors (*p* = 0.135).

**FIGURE 4:**
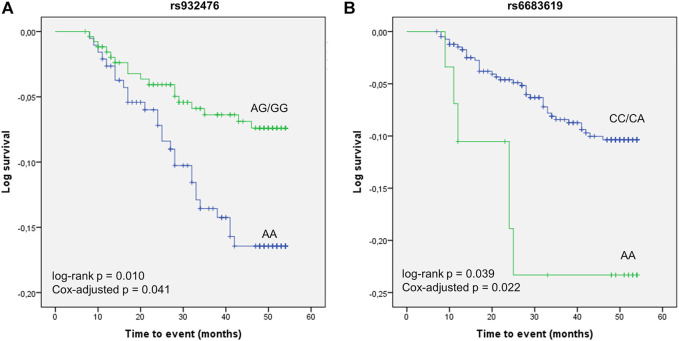
Kaplan-Meier curves of the association of two *PLA2G4A* polymorphisms, rs932476 **(A)** and rs6683619 **(B)**, with CV event-free survival.

We next examined associations with CV event-free survival in the cohort of nephrosclerosis patients. Eight SNPs, all in the *PLA2G4A* gene, displayed log-rank *p*-values <0.1 and were subsequently subjected to Cox regression analysis. After adjusting for other CV risk factors, the AA genotype of rs932476 significantly decreased CV event-free survival [estimated mean = 49.59 (47.97–51.21) vs. 51.81 (49.93–51.78) months for AG/GG carriers, *p* = 0.041, Figure 4A]. In addition, carriers of the rs6683619 AA genotype also had lower survival than subjects harbouring the CC/CA genotypes [46.46 (41.00–51.92) vs. 51.17 (50.25–52.08) months, *p* = 0.022, Figure 4B].

## Discussion

There is a need to find new biomarkers in the field of CKD that can help identify patients at risk and that are able to predict how fast the disease is going to progress to ESRD. This need is pressing in nephrosclerosis, as these patients often find themselves within a vicious circle, where kidney deterioration worsens CV risk whilst, in turn, these CV implications accelerate disease progression. To our knowledge, this is the first time that the clinical impact of phospholipase-related SNPs has been evaluated in the nephrosclerosis setting, which is ideal for investigating atherosclerosis measurements and CV outcomes. Indeed, nephrosclerosis and atherosclerosis go frequently hand in hand (Kawamoto et al., 2008; Erten et al., 2011). Furthermore, nephrosclerosis patients suffer from a CV mortality which is up to 20 times more frequent than in the general population (Foley et al., 1998) and have mortality rates higher than other CKD groups after renal replacement therapy (4).

Our results showed that two SNPs in the *SCARB1* gene, rs10846744, and rs838880, were significantly linked to the susceptibility to nephrosclerosis. The SR-BI receptor, encoded by *SCARB1*, is the primary mediator of the selective transfer of lipids from HDL into cells, and has also been shown to mediate the internalization of oxidized phospholipids (Gillotte-Taylor et al., 2001). In contrast to native HDL, oxidized HDL may increase the production of inflammatory cytokines. In this regard, oxidized HDL has been reported to enhance proinflammatory properties in mesangial and other renal cells, which has been pointed out as an important mechanism of CKD (Zhang et al., 2010; Gao et al., 2014). Moreover, SR-BI expression has been shown to be increased in animal models of chronic renal failure (Cho et al., 2010). It is therefore tempting to speculate that genetic variants affecting SR-B1 activity/expression could increase the transport of oxidized lipids into renal cells and hence constitute an explanation for the observed association with the increased nephrosclerosis risk. Other mechanisms are also possible. For instance, rs10846744 is a widely studied variant located in intron 1 of the *SCARB1* gene containing DNase I hypersensitivity clusters and enhancer-promoter histone markers. This SNP has not shown correlation with SR-BI expression (Manichaikul et al., 2012), rather, it has been proposed to mediate the enhanced expression of distant genes affecting apoptosis (Li et al., 2005) and endothelial (Mineo and Shaul, 2007) or inflammatory pathways (Suchindran et al., 2010), which could also contribute to the disease. Three variants in *PLA2G4A* also showed associations with the risk of nephrosclerosis, although rs72709847 and rs1569479 were at the border of statistical significance. The remaining tag-SNP, rs78178583, is an intronic A-to-C substitution located within the promoter flanking region of the gene (ENSR00000934514), and therefore it is plausible that it could be tagging genetic variability modulating cPLA2 expression, which could in turn affect the risk of CKD, since cPLA2 expression has been related to renal damage both in humans and animal models (Montford et al., 2016). A suggested mechanism would be that this enzyme releases AA from the membrane leading to the synthesis of proinflammatory mediators such as PGE2, a major actor mediating renal injury (Francois et al., 2007).

Nephrosclerosis and atherosclerosis share risk factors and similar etiopathogenic mechanisms (Meyrier, 1996). Renal parenchyma is replaced by collagen and scar tissue in the former, whilst the latter presents with eccentric and medial thickening of arteries. Indeed, it has been proposed that increased atherosclerosis contribute to the rate of glomerulosclerosis observed in these CKD patients (Tracy et al., 1996). In this regard, a Japanese study had reported an inverse correlation between ccIMT and eGFR values (Kawamoto et al., 2008), which we could also observe in our population and that it might support the connection between renal and CV disease. However, the observed correlation, although significant, was very weak and should be interpreted with caution. Our findings also show that variability in the *PLA2G4A* and *PLA2G7* gene loci was associated with several atherosclerotic features in nephrosclerosis patients, including the risk of an accelerated progression. Whilst there is no available information on the effect of *PLA2G4A* variants on atherosclerosis measurements, there are some previous reports linking *PLA2G7* SNPs to this pathology. Thus, (Wu et al., 2020) reported that the A379V polymorphism was associated with carotid plaque formation, but not plaque vulnerability, in ischemic stroke patients . In the same line, (Santoso et al., 2017) performed a meta-analysis including over 12,000 patients and identified two nonsynonymous SNPs significantly associated with the risk of clinical atherosclerosis . The results presented herein confirm the important role of phospholipases in atherosclerosis, highlight *PLA2G4A* as a genetic locus to be considered, and overall point at nephrosclerosis as an atherosclerosis-related pathology where the screening of phospholipase genes could be useful to identify patients more prone to a worse clinical course.

A remarkable finding of this study was that several tag-SNPs in *PLA2G4A* were independent CV risk factors in the nephrosclerosis patients. Moreover, they significantly enhanced the predictive power of a model containing standard, non-genetic risk factors. It should be noticed how the improvement after adding the genetic information was greater in the nephrosclerosis patients than in the whole study sample, as the incidence of classic risk factors in the former is already high. It is somewhat surprising that it was the *PLA2G4A* gene, and not *PLA2G7*, coding for the traditionally CVD-associated Lp-PLA2, that exerted the most noticeable influence on the incidence of CV events. However, it should be noted that the association of *PLA2G7* with CVD is still controversial (Hou et al., 2009; Casas et al., 2010; Zheng et al., 2011; Garg et al., 2018). For instance, a large study with over 45,000 coronary heart disease patients and 88,000 controls could not find any loss-of-function allele related to disease risk (Gregson et al., 2017). Likewise, clinical trials with darapladib (Lp-PLA2 inhibitor) have failed to show benefit on the prevention of major coronary events (White et al., 2014; O'Donoghue et al., 2014). On the other hand, it could also be that the CV risk genetic profile in nephrosclerosis patients was different from that occurring in the general population. Unfortunately, only eight events were registered in our control subjects and hence no formal analysis could be carried out to address this hypothesis.

The results from the survival study also pointed to *PLA2G4A* as a key genetic locus regarding CV risk in nephrosclerosis, with two tag-SNPs (rs932476 and rs6683619) significantly affecting event-free survival. In any case, it should be mentioned that the association with rs6683619 was only observed for a recessive model and hence it was based on a low number of events. There is a growing body of evidence supporting the involvement of *PLA2G4A* in CVD. The main function of cPLA2, the enzyme encoded by *PLA2G4A*, is the release of arachidonic acid from the membrane to initiate a cascade of pro-inflammatory mediators. In this regard, our group has previously reported an association between SNPs in PGE2 receptors, at the end of this cascade, with the occurrence of CV events in CKD patients (González et al., 2021). In addition, cPLA2 have been shown to mediate platelet aggregation induced by homocysteinemia (d'Emmanuele di Villa Bianca et al., 2013) and by high doses of digoxin in atrial fibrillation that can be behind CV mortality in these patients (Pastori et al., 2018). Moreover, polymorphisms in *PLA2G4A* have also been related to myocardial infarction (Hartiala et al., 2012) and coronary artery disease (Hartiala et al., 2011). Overall, our results are in line with this background and indicate that the areas of *PLA2G4A* tagged by the SNPs presented herein hold the potential to be useful biomarkers for CV risk in nephrosclerosis patients. Furthermore, these findings suggest that cPLA2 may be an interesting therapeutic target and that novel cPLA2 inhibitors that are being currently developed for a variety of inflammatory diseases (Nikolaou et al., 2019) should also be evaluated for CVD.

In our cohort, significant sex-related differences were observed regarding the incidence of nephrosclerosis and the occurrence of CV events, with males showing higher risk for both outcomes. To establish whether the studied SNPs could, at least in part, be behind these differences, we analyzed their distribution both in CKD patients and in the whole population (data not shown). None of the SNPs with a relevant role in the risk of nephrosclerosis or in CV outcomes were differently distributed between men and women. Therefore, we found no evidence supporting a role of the studied variants in the observed sex-related differences in nephrosclerosis.

Our study has several limitations. First, we did not measure Lp-PLA2 activity, which has been related to CV outcomes (Huang et al., 2020) and could have helped identify putative mechanisms for the genotype-phenotype associations reported. Second, the *PLA2G4A* gene was far more polymorphic than *PLA2G7 and SCARB1* and consequently far more SNPs were necessary to tag the whole locus, which could have led to the obtention of more significant results. Third, our study design implies that the reported genotype-phenotype associations cannot, in general, be linked to a specific biochemical consequence of the SNP, as these were tag-SNPs, i.e., intronic variants that represent variability in a certain haplotype block. In contrast, one of the strengths of the study is precisely that by revealing clinically relevant tag-SNPs, we made it possible to infer total genetic variability in the tagged region of the gene locus and identify phenotypic associations without having to determine the rest of SNPs in that area. Another asset of the study was the uncommon homogeneity of the patient cohort, as CKD diagnosis was limited to nephrosclerosis, thus excluding patients with diabetic nephropathy, who are usually included together with nephrosclerosis patients in a single entity (Smith et al., 2020).

In summary, our results taken together indicate that phospholipase-related genes play a relevant role in nephrosclerosis and associated CV outcomes. We showed that *SCARB1* for the risk of nephrosclerosis, *PLA2G7* and, especially, *PLA2G4A* for the CV risk in these patients, are loci that harbor genetic variants whose identification could be of utility in the management of this CKD.

## Data Availability

The datasets presented in this study can be found in online repositories. The names of the repository/repositories and accession number(s) can be found below: https://figshare.com/, https://figshare.com/articles/dataset/Phospholipases_genes_dataset/17032403.
