# Supplementary material for: Tag-SNPs in Phospholipase-Related Genes Modify the Susceptibility to Nephrosclerosis and its Associated Cardiovascular Risk
Source: Front Pharmacol. 2022 May 2;13:817020. doi: 10.3389/fphar.2022.817020 (PMC9108153; doi:10.3389/fphar.2022.817020)
Supplement: Supplementary file 1 [file Table1.DOCX]

**SUPPLEMENTARY TABLES**

**Supplementary Table S1.** Genetic polymorphisms included in the study

| **rs number** | **Gene** | **Position** | **Reference allele** | **Alternate allele** | **HWE** |
| --- | --- | --- | --- | --- | --- |
| rs9472817 | *PLA2G7* | 6:46673255 | G | C | 0.735 |
| rs41273658 | *PLA2G7* | 6:46688820 | G | A | 0.906 |
| rs1051931 | *PLA2G7* | 6:46705206 | A | G | 0.351 |
| rs2216465 | *PLA2G7* | 6:46708282 | T | C | 1.000 |
| rs6899519 | *PLA2G7* | 6:46714163 | G | A | 0.365 |
| rs1362931 | *PLA2G7* | 6:46714342 | G | C | 0.952 |
| rs17288905 | *PLA2G7* | 6:46715645 | G | A | 0.510 |
| rs16874962 | *PLA2G7* | 6:46715740 | C | T | 0.751 |
| rs12195701 | *PLA2G7* | 6:46719682 | C | T | 0.330 |
| rs2216463 | *PLA2G7* | 6:46720992 | T | G | 0.758 |
| rs9472831 | *PLA2G7* | 6:46722110 | A | G | 0.618 |
| rs12528807 | *PLA2G7* | 6:46728770 | G | A | 0.162 |
| rs1421368 | *PLA2G7* | 6:46728856 | T | G | 0.207 |
| rs1421369 | *PLA2G7* | 6:46732969 | G | A | 0.699 |
| rs9395208 | *PLA2G7* | 6:46735582 | A | G | 0.945 |
| rs6915496 | *PLA2G7* | 6:46741875 | C | G | 0.600 |
| rs9472836 | *PLA2G7* | 6:46742987 | C | G | 0.400 |
| rs838880 | *SCARB1* | 12:124777047 | C | T | 0.305 |
| rs5888 | *SCARB1* | 12:124800202 | C | T | 1.000 |
| rs2278986 | *SCARB1* | 12:124814823 | G | A | 1.000 |
| rs11057830 | *SCARB1* | 12:124822507 | C | A | 1.000 |
| rs10846744 | *SCARB1* | 12:124827879 | G | A | 0.156 |
| rs12064238 | *PLA2G4A* | 1:186689993 | T | A | 0.314 |
| rs10911924 | *PLA2G4A* | 1:186808246 | C | T | **<0.001** |
| rs979924 | *PLA2G4A* | 1:186828347 | C | A | 1.000 |
| rs12097836 | *PLA2G4A* | 1:186830940 | G | A | 0.386 |
| rs78099806 | *PLA2G4A* | 1:186833089 | G | T | 0.160 |
| rs7535459 | *PLA2G4A* | 1:186833431 | T | C | 0.337 |
| rs35180943 | *PLA2G4A* | 1:186833659 | A | G | 0.244 |
| rs60292300 | *PLA2G4A* | 1:186836921 | A | G | 0.679 |
| rs3820185 | *PLA2G4A* | 1:186839211 | T | A | 0.405 |
| rs78178583 | *PLA2G4A* | 1:186843860 | A | G | 0.704 |
| rs10752976 | *PLA2G4A* | 1:186852221 | A | C | 0.726 |
| rs12070719 | *PLA2G4A* | 1:186853015 | C | T | **0.001** |
| rs10911930 | *PLA2G4A* | 1:186853238 | A | G | 0.840 |
| rs12070830 | *PLA2G4A* | 1:186853262 | A | T | 0.354 |
| rs2076075 | *PLA2G4A* | 1:186854152 | T | A | 0.877 |
| rs763419 | *PLA2G4A* | 1:186854826 | G | C | 0.413 |
| rs2223305 | *PLA2G4A* | 1:186855707 | G | T | 0.488 |
| rs6696406 | *PLA2G4A* | 1:186858985 | T | C | 0.780 |
| rs12404877 | *PLA2G4A* | 1:186860960 | G | T | 0.247 |
| rs12747953 | *PLA2G4A* | 1:186864564 | T | C | **<0.001** |
| rs77897442 | *PLA2G4A* | 1:186865541 | C | G | 1.000 |
| rs10911937 | *PLA2G4A* | 1:186866005 | C | A | 0.298 |
| rs7532616 | *PLA2G4A* | 1:186867757 | T | C | 0.836 |
| rs12567135 | *PLA2G4A* | 1:186869201 | G | T | 0.394 |
| rs7547993 | *PLA2G4A* | 1:186870729 | A | G | 0.939 |
| rs72707570 | *PLA2G4A* | 1:186872829 | C | T | 1.000 |
| rs10578509 | *PLA2G4A* | 1:186873127 | C | T | 0.085 |
| rs2223307 | *PLA2G4A* | 1:186875794 | T | C | 0.434 |
| rs75393128 | *PLA2G4A* | 1:186876336 | A | G | 0.311 |
| rs17591849 | *PLA2G4A* | 1:186878442 | T | G | 0.172 |
| rs12746200 | *PLA2G4A* | 1:186880054 | C | T | 0.231 |
| rs1980444 | *PLA2G4A* | 1:186881828 | G | C | 0.827 |
| rs17526478 | *PLA2G4A* | 1:186882025 | G | A | 0.844 |
| rs2049963 | *PLA2G4A* | 1:186882084 | A | G | 0.215 |
| rs35174355 | *PLA2G4A* | 1:186885413 | T | C | 0.769 |
| rs2064115 | *PLA2G4A* | 1:186885833 | A | T | **0.046** |
| rs10911946 | *PLA2G4A* | 1:186886074 | C | T | 0.609 |
| rs12749354 | *PLA2G4A* | 1:186887116 | T | A | 0.615 |
| rs2205898 | *PLA2G4A* | 1:186888827 | A | C | 0.486 |
| rs11577826 | *PLA2G4A* | 1:186889374 | A | T | 0.594 |
| rs72709847 | *PLA2G4A* | 1:186890198 | G | C | 0.731 |
| rs12143166 | *PLA2G4A* | 1:186890357 | A | G | **0.044** |
| rs6656909 | *PLA2G4A* | 1:186904098 | T | G | 0.589 |
| rs1569479 | *PLA2G4A* | 1:186904572 | T | G | 0.381 |
| rs61810980 | *PLA2G4A* | 1:186905233 | A | C | 0.669 |
| rs12720557 | *PLA2G4A* | 1:186908798 | A | G | 0.650 |
| rs10911958 | *PLA2G4A* | 1:186921713 | G | A | 0.548 |
| rs112568781 | *PLA2G4A* | 1:186937435 | C | G | 0.245 |
| rs6683619 | *PLA2G4A* | 1:186944870 | A | G | 0.508 |
| rs7514702 | *PLA2G4A* | 1:186944891 | C | T | 0.337 |
| rs11587539 | *PLA2G4A* | 1:186946045 | G | A | 0.068 |
| rs10911967 | *PLA2G4A* | 1:186947614 | A | G | 0.507 |
| rs969358 | *PLA2G4A* | 1:186951060 | T | del | 0.666 |
| rs4276871 | *PLA2G4A* | 1:186960581 | C | T | 0.445 |
| rs61208933 | *PLA2G4A* | 1:186962045 | C | T | **0.043** |
| rs10798075 | *PLA2G4A* | 1:186963079 | A | G | 0.248 |
| rs932476 | *PLA2G4A* | 1:186964423 | A | G | 0.297 |
| rs2383556 | *PLA2G4A* | 1:186966509 | C | T | 1.000 |
| rs4650714 | *PLA2G4A* | 1:186973681 | G | A | 1.000 |
| rs12131535 | *PLA2G4A* | 1:186976365 | T | C | 0.914 |
| rs4402086 | *PLA2G4A* | 1:186983277 | G | A | 0.680 |
| rs10752989 | *PLA2G4A* | 1:186986298 | C | T | 0.903 |
| rs6682706 | *PLA2G4A* | 1:186987081 | A | G | 0.241 |
| rs1555204 | *PLA2G4A* | 1:186988083 | C | G | 0.184 |

HWE, Hardy-Weinberg equilibrium

**Supplementary Table S2.** P-values for the association of SCARB1 and PLA2G7 tag-SNPs with renal function (estimated glomerular filtration rate, eGFR) and damage (albumin-to-creatinine ratio).

|  |  | **eGFR** | | | **Albumin-to-cratinine ratio** | | |
| --- | --- | --- | --- | --- | --- | --- | --- |
| **Gene** | **rs number** | **dominant model** | **recessive model** | **Log-additive model** | **dominant model** | **recessive model** | **Log-additive model** |
| *SCARB1* | rs838880 | 0.775 | 0.345 | 0.525 | 0.235 | 0.845 | 0.322 |
| *SCARB1* | rs5888 | **0.021** | 0.104 | **0.015** | 0.670 | 0.347 | 0.813 |
| *SCARB1* | rs2278986 | 0.481 | 0.684 | 0.471 | 0.226 | 0.505 | 0.217 |
| *SCARB1* | rs11057830 | 0.959 | **0.021** | 0.479 | 0.732 | 0.848 | 0.818 |
| *SCARB1* | rs10846744 | 0.914 | **0.033** | 0.395 | 0.717 | 0.886 | 0.722 |
| *PLA2G7* | rs9472817 | 0.701 | 0.501 | 0.529 | 0.345 | 0.160 | 0.154 |
| *PLA2G7* | rs41273658 | 0.472 | - | - | 0.590 | - | - |
| *PLA2G7* | rs1051931 | 0.702 | 0.451 | 0.994 | 0.247 | 0.892 | 0.317 |
| *PLA2G7* | rs2216465 | 0.821 | **0.008** | 0.184 | 0.621 | 0.731 | 0.589 |
| *PLA2G7* | rs6899519 | 0.532 | **0.041** | 0.208 | 0.153 | 0.064 | 0.063 |
| *PLA2G7* | rs1362931 | 0.715 | 0.290 | 0.478 | 0.129 | 0.997 | 0.212 |
| *PLA2G7* | rs17288905 | 0.536 | 0.223 | 0.442 | 0.400 | - | - |
| *PLA2G7* | rs16874962 | 0.539 | 0.490 | 0.652 | 0.935 | 0.591 | 0.960 |
| *PLA2G7* | rs12195701 | 0.105 | 0.079 | **0.048** | 0.382 | **0.043** | 0.149 |
| *PLA2G7* | rs2216463 | 0.572 | 0.591 | 0.842 | 0.473 | 0.670 | 0.693 |
| *PLA2G7* | rs9472831 | 0.420 | 0.243 | 0.235 | 0.246 | 0.101 | 0.088 |
| *PLA2G7* | rs12528807 | 0.602 | 0.402 | 0.499 | 0.092 | 0.508 | 0.097 |
| *PLA2G7* | rs1421368 | 0.626 | 0.903 | 0.705 | 0.433 | 0.633 | 0.412 |
| *PLA2G7* | rs1421369 | 0.881 | **0.012** | 0.143 | 0.195 | 0.924 | 0.374 |
| *PLA2G7* | rs9395208 | 0.229 | 0.138 | 0.132 | **0.029** | 0.898 | 0.066 |
| *PLA2G7* | rs6915496 | 0.113 | 0.910 | 0.128 | 0.347 | 0.329 | 0.282 |
| *PLA2G7* | rs9472836 | 0.699 | 0.552 | 0.887 | 0.121 | 0.215 | 0.378 |

**Supplementary Table S3.** P-values for the association between tag-SNPs in SCARB1, PLA2G7 and PLA2G4A with the incidence of cardiovascular events in a four-year follow-up. Results using dominant, recessive and additive models are shown.

| **Gene** | **rs number** | **Dominant Model** | **Recessive Model** | **Log-additive Model** |
| --- | --- | --- | --- | --- |
| *SCARB1* | rs838880 | 0.431 | 0.605 | 0.408 |
| *SCARB1* | rs5888 | 0.675 | 0.845 | 0.700 |
| *SCARB1* | rs2278986 | 0.490 | 0.470 | 0.394 |
| *SCARB1* | rs11057830 | 0.261 | 0.600 | 0.252 |
| *SCARB1* | rs10846744 | 0.241 | 0.395 | 0.196 |
| *PLA2G7* | rs9472817 | 0.785 | 0.573 | 0.909 |
| *PLA2G7* | rs41273658 | 0.413 | - | - |
| *PLA2G7* | rs1051931 | 0.563 | 0.252 | 0.995 |
| *PLA2G7* | rs2216465 | 0.902 | 0.585 | 0.887 |
| *PLA2G7* | rs6899519 | 0.251 | 0.295 | 0.552 |
| *PLA2G7* | rs1362931 | 0.427 | 0.182 | 0.919 |
| *PLA2G7* | rs17288905 | 0.599 | 0.597 | 0.655 |
| *PLA2G7* | rs16874962 | 0.851 | 0.494 | 0.939 |
| *PLA2G7* | rs12195701 | 0.334 | 0.880 | 0.367 |
| *PLA2G7* | rs2216463 | 0.974 | 0.490 | 0.769 |
| *PLA2G7* | rs9472831 | 0.526 | 0.815 | 0.778 |
| *PLA2G7* | rs12528807 | 0.261 | 0.252 | 0.503 |
| *PLA2G7* | rs1421368 | 0.216 | 0.074 | 0.716 |
| *PLA2G7* | rs1421369 | 0.694 | 0.646 | 0.988 |
| *PLA2G7* | rs9395208 | 0.159 | 0.881 | 0.195 |
| *PLA2G7* | rs6915496 | 0.080 | 0.426 | 0.124 |
| *PLA2G7* | rs9472836 | 0.743 | 0.582 | 0.637 |
| *PLA2G4A* | rs12064238 | 0.328 | 0.248 | 0.216 |
| *PLA2G4A* | rs10911924 | 0.694 | 0.827 | 0.688 |
| *PLA2G4A* | rs979924 | 0.427 | 0.651 | 0.469 |
| *PLA2G4A* | rs12097836 | 0.548 | **0.046** | 0.174 |
| *PLA2G4A* | rs78099806 | 0.311 | 0.682 | 0.352 |
| *PLA2G4A* | rs7535459 | 0.753 | 0.365 | 0.503 |
| *PLA2G4A* | rs35180943 | 0.468 | **0.029** | 0.217 |
| *PLA2G4A* | rs60292300 | 0.920 | 0.673 | 0.882 |
| *PLA2G4A* | rs3820185 | 0.925 | **0.019** | 0.296 |
| *PLA2G4A* | rs78178583 | 0.763 | 0.574 | 0.820 |
| *PLA2G4A* | rs10752976 | 0.814 | 0.159 | 0.731 |
| *PLA2G4A* | rs12070719 | 0.594 | 0.349 | 0.724 |
| *PLA2G4A* | rs10911930 | 0.507 | 0.253 | 0.809 |
| *PLA2G4A* | rs12070830 | 0.299 | 0.116 | 0.582 |
| *PLA2G4A* | rs2076075 | 0.650 | 0.153 | 0.461 |
| *PLA2G4A* | rs763419 | 0.708 | 0.172 | 0.971 |
| *PLA2G4A* | rs2223305 | 0.091 | 0.373 | 0.121 |
| *PLA2G4A* | rs6696406 | 0.919 | 0.147 | 0.637 |
| *PLA2G4A* | rs12404877 | 0.808 | 0.804 | 0.771 |
| *PLA2G4A* | rs12747953 | 0.860 | **0.043** | 0.617 |
| *PLA2G4A* | rs77897442 | 0.445 | 0.479 | 0.401 |
| *PLA2G4A* | rs10911937 | 0.594 | 0.477 | 0.926 |
| *PLA2G4A* | rs7532616 | 0.340 | 0.107 | 0.217 |
| *PLA2G4A* | rs12567135 | 0.523 | 0.346 | 0.350 |
| *PLA2G4A* | rs7547993 | 0.743 | **0.025** | 0.708 |
| *PLA2G4A* | rs72707570 | 0.125 | 0.720 | 0.394 |
| *PLA2G4A* | rs10578509 | 0.930 | 0.374 | 0.824 |
| *PLA2G4A* | rs2223307 | 0.615 | **0.049** | 0.935 |
| *PLA2G4A* | rs75393128 | 0.266 | 0.804 | 0.278 |
| *PLA2G4A* | rs17591849 | 0.262 | - | - |
| *PLA2G4A* | rs12746200 | 0.487 | 0.451 | 0.438 |
| *PLA2G4A* | rs1980444 | 0.960 | 0.164 | 0.727 |
| *PLA2G4A* | rs17526478 | 0.995 | **0.007** | 0.380 |
| *PLA2G4A* | rs2049963 | 0.407 | 0.298 | 0.331 |
| *PLA2G4A* | rs35174355 | 0.749 | 0.244 | 0.608 |
| *PLA2G4A* | rs2064115 | 0.959 | 0.538 | 0.897 |
| *PLA2G4A* | rs10911946 | 0.742 | **0.002** | 0.394 |
| *PLA2G4A* | rs12749354 | 0.613 | 0.246 | 0.491 |
| *PLA2G4A* | rs2205898 | 0.969 | 0.126 | 0.698 |
| *PLA2G4A* | rs11577826 | 0.771 | 0.077 | 0.644 |
| *PLA2G4A* | rs72709847 | 0.185 | 0.512 | 0.171 |
| *PLA2G4A* | rs12143166 | 0.750 | 0.141 | 0.369 |
| *PLA2G4A* | rs6656909 | 0.583 | 0.328 | 0.814 |
| *PLA2G4A* | rs1569479 | 0.199 | 0.052 | 0.058 |
| *PLA2G4A* | rs61810980 | 0.594 | 0.363 | 0.807 |
| *PLA2G4A* | rs12720557 | 0.267 | 0.460 | 0.230 |
| *PLA2G4A* | rs10911958 | 0.917 | 0.897 | 0.888 |
| *PLA2G4A* | rs112568781 | **0.018** | 0.184 | **0.014** |
| *PLA2G4A* | rs6683619 | 0.119 | 0.103 | 0.590 |
| *PLA2G4A* | rs7514702 | 0.201 | 0.939 | 0.294 |
| *PLA2G4A* | rs11587539 | 0.732 | 0.897 | 0.865 |
| *PLA2G4A* | rs10911967 | 0.605 | 0.124 | 0.271 |
| *PLA2G4A* | rs969358 | 0.180 | - | - |
| *PLA2G4A* | rs4276871 | 0.574 | 0.902 | 0.647 |
| *PLA2G4A* | rs61208933 | 0.084 | 0.799 | 0.157 |
| *PLA2G4A* | rs10798075 | 0.263 | 0.309 | 0.197 |
| *PLA2G4A* | rs932476 | 0.081 | 0.312 | 0.075 |
| *PLA2G4A* | rs2383556 | 0.187 | 0.368 | 0.161 |
| *PLA2G4A* | rs4650714 | 0.365 | - | - |
| *PLA2G4A* | rs12131535 | 0.586 | 0.645 | 0.539 |
| *PLA2G4A* | rs4402086 | 0.298 | 0.897 | 0.365 |
| *PLA2G4A* | rs10752989 | 0.387 | 0.677 | 0.409 |
| *PLA2G4A* | rs6682706 | 0.563 | 0.602 | 0.715 |
| *PLA2G4A* | rs1555204 | 0.359 | 0.517 | 0.324 |
