# Supplementary figures and images for: Tag-SNPs in Phospholipase-Related Genes Modify the Susceptibility to Nephrosclerosis and its Associated Cardiovascular Risk
Source: Front Pharmacol. 2022 May 2;13:817020. doi: 10.3389/fphar.2022.817020 (PMC9108153; doi:10.3389/fphar.2022.817020)

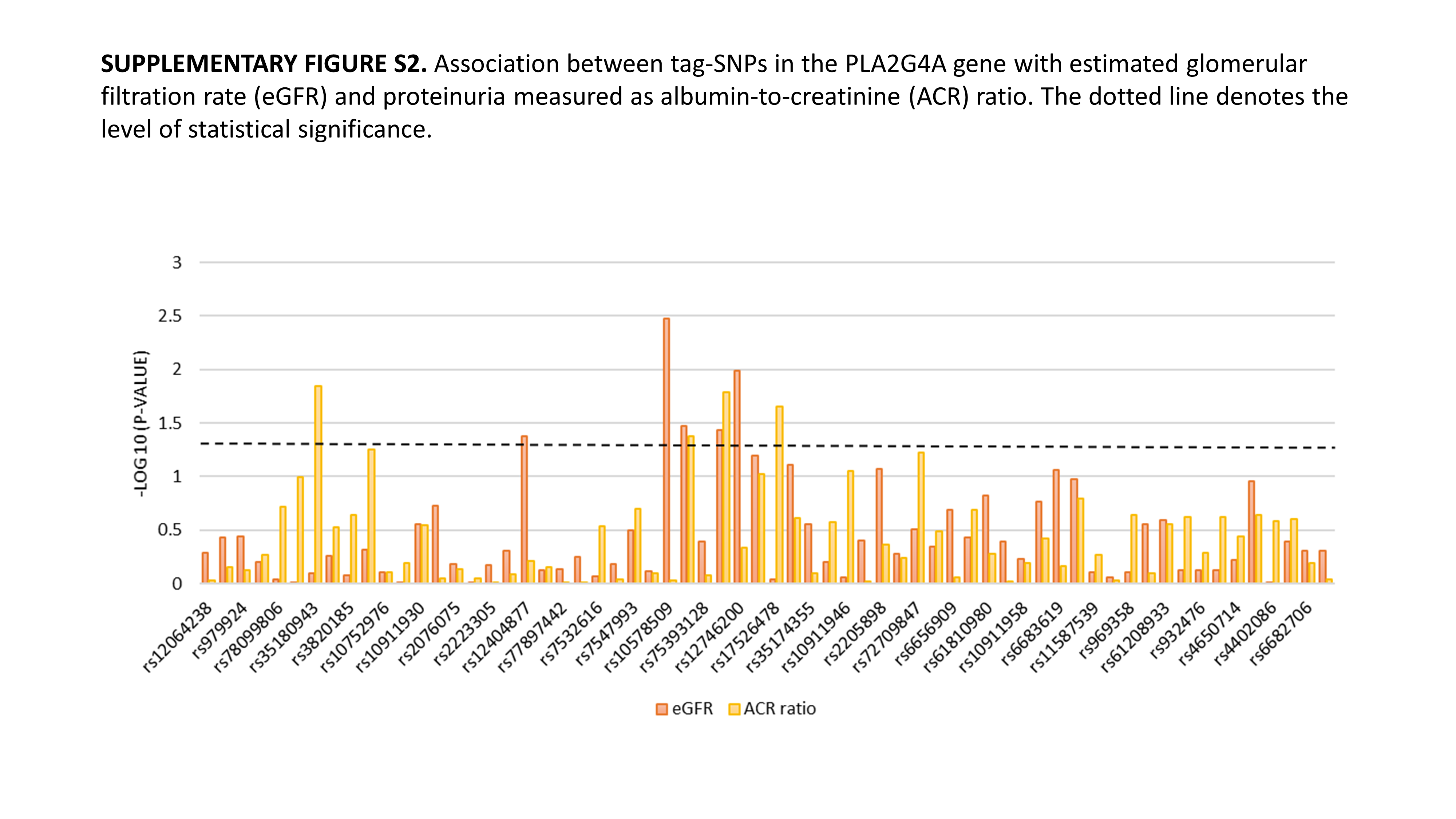

Supplement: Supplementary file 2 [file Image2.TIF]

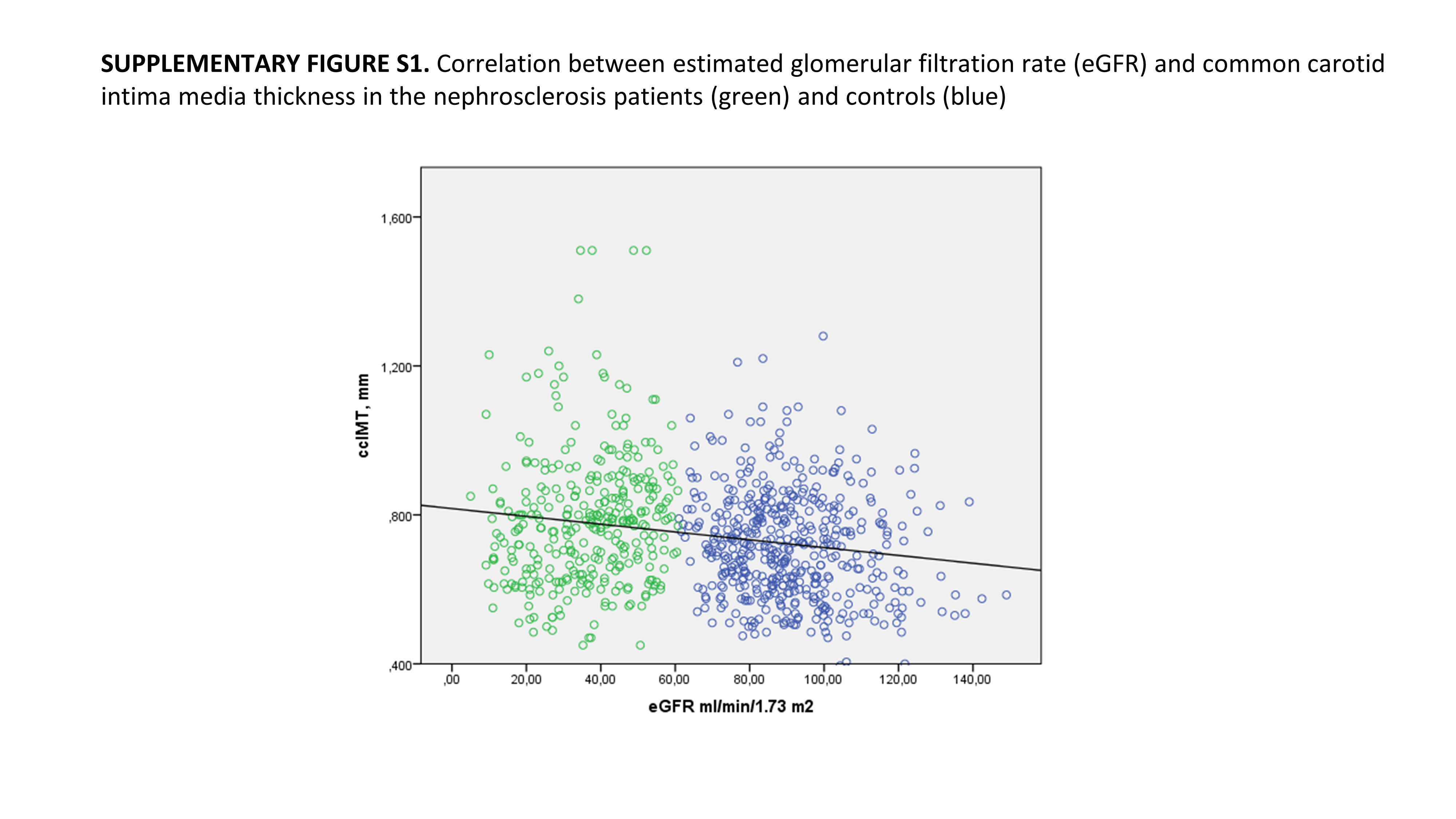

Supplement: Supplementary file 3 [file Image1.TIF]
